# Assessing Rotational Ankle Instability Through Postural Control Testing: Coronal Instability Outperforms Conventional Imaging

**DOI:** 10.1002/jfa2.70091

**Published:** 2025-10-21

**Authors:** Nan Mei, Zhende Jiang, Zhuan Zhong, Yaokuan Ruan, Hengyu Liu, Hiroaki Kurokawa, Takuma Miyamoto, Akira Taniguchi, Yasuhito Tanaka, Fei Chang

**Affiliations:** ^1^ Orthopaedic Medical Center The Second Hospital of Jilin University Changchun China; ^2^ Department of Orthopaedic Surgery Nara Medical University Nara Japan; ^3^ Jilin Sport University Changchun China

**Keywords:** chronic ankle instability, deltoid ligament, diagnostic imaging, postural control, rotational ankle instability

## Abstract

**Background:**

Deltoid ligament (DL) injuries are increasingly recognized in chronic ankle instability (CAI), drawing clinical attention to rotational ankle instability (RAI). Cadaveric studies have shown that RAI can increase ankle rotation; however, current examination methods for RAI remain limited. As they neither provide adequate insight into ligamentous structural damage nor clearly characterize the rotational instability. This study aimed to evaluate the characteristic rotational instability of RAI and, based on this indicator, investigate which postural control parameters best represent rotational instability and how these parameters can be quantified to assess diagnostic utility using postural control parameters and establish quantitative diagnostic thresholds.

**Methods:**

We included 32 patients with CAI who underwent a postural control assessment, combining center of pressure (COP) analysis with the sensory organization test (SOT). Talar tilt angle and medial clear space were also measured via anteroposterior radiographs and magnetic resonance imaging (MRI) to assess DL injuries. Final diagnoses of RAI or CAI were made in the DL based on arthroscopic findings. Receiver operating characteristic (ROC) curves were constructed to determine the diagnostic performance of each indicator.

**Results:**

In RAI, the ratio of coronal‐plane sway to sagittal‐plane sway amplitude during motion was significantly elevated, a phenomenon we term “coronal instability.” Coronal instability emerged as a strong predictor of RAI, with an area under the ROC curve (AUC) of 0.95 (95% CI, 0.810–0.996; *p* < 0.0001). Its optimal cutoff value of 0.81 yielded a sensitivity of 83.33% and a specificity of 100%, surpassing imaging‐based measures such as radiography and MRI (AUC = 0.567–0.844).

**Conclusion:**

Coronal instability, measured through a noninvasive postural control assessment, demonstrates high sensitivity and specificity for diagnosing RAI. This method offers a valuable clinical tool for accurately identifying RAI and may complement or outperform traditional imaging techniques in certain cases.

## 1 Introduction

Rotational ankle instability (RAI) has gained increasing attention in patients with chronic ankle instability (CAI) [1, 2, 3]. RAI is characterized by an increased rotational component at the ankle, often involving injuries to the deltoid ligament (DL) and lateral ligaments, such as the anterior talofibular ligament (ATFL) [1, 2]. It has been reported that the incidence of RAI in patients with CAI ranges from 36% to 72%, suggesting that a significant proportion of patients with CAI might have underlying rotational components [4, 5, 6, 7]. Failure to identify and address DL injuries associated with RAI can lead to persistent postoperative instability [1, 8].

Despite growing interest in RAI, there is no universally accepted clinical or imaging‐based diagnostic criterion specifically for rotational instability. Traditional imaging modalities, including X‐ray and MRI, have demonstrated moderate‐to‐high reliability in detecting ligamentous damage but may not adequately capture dynamic rotational components [5, 9, 10, 11]. Additionally, although many clinicians utilize stress radiographs or clinical examination to evaluate the medial clear space for DL injuries, these methods lack definitive sensitivity and specificity for diagnosing RAI. Computed tomography (CT) can offer detailed bone structure imaging and Hounsfield unit (HU) values for potential assessment of ligament degeneration, but standardized thresholds for diagnosing DL damage remain unclear [7]. Therefore, static imaging assessments appear to have limitations in capturing dynamic rotational instability [6, 10, 12]. In cases of CAI where surgery is indicated, arthroscopy can help directly visualize “open book” sign suggestive of DL injury [1, 2, 13]. However, although arthroscopy is minimally invasive and can be both diagnostic and therapeutic, it is not typically performed solely for diagnostic purposes in mild or uncertain cases of instability.

Recent biomechanical and cadaveric studies have highlighted the critical role of the DL in maintaining rotational stability; injury to these components can significantly compromise the ankle's rotational integrity [3, 13, 14, 15]. Specifically, center of pressure (COP) sway parameters and the Sensory Organization Test (SOT) can provide quantitative data on postural balance and dynamic stability [16, 17, 18, 19, 20]. These metrics have shown high reliability (ICC, 0.70–0.95) and have been applied to evaluate and monitor rehabilitation outcomes in various ankle and lower‐limb pathologies, including CAI and syndesmotic injuries [21, 22]. However, their utility in diagnosing the rotational aspect of ankle instability has yet to be fully validated [16, 17].

Against this background, the purpose of this study was to investigate whether COP and SOT parameters can serve as highly sensitive and specific indicators for identifying RAI. By comparing postural control‐based measurements with arthroscopic findings and conventional imaging parameters, we sought to determine an optimal threshold that correlates with RAI diagnosis. We hypothesize that postural control measures, particularly those reflecting coronal‐plane sway, will outperform traditional static imaging in detecting rotational instability. Our ultimate aim is to establish a clinically feasible noninvasive method that can accurately distinguish RAI from more generalized CAI, thereby improving diagnostic precision and guiding more targeted surgical or conservative interventions.

## 2 Materials and Methods

### 2.1 Study Design and Participants

This cross‐sectional study was conducted at both the Foot and Ankle Surgery Department of a local hospital and the Training and Therapy Laboratory of a university. Patients diagnosed with CAI were included consecutively between December 2021 and June 2024 [23]. All participants provided written informed consent, and the study protocol was approved by the institutional ethics committee (No. 2021130).

Participant inclusion criteria for CAI are as follows: (1) at least one severe ankle sprain in the year prior to the examination, (2) ankle instability, giving away or recurrent sprain sensation, and (3) a score of < 24 on the Cumberland Ankle Instability Scale.

Exclusion criteria are as follows: (1) A history of lower limb fractures or surgery, (2) ankle sprain of < 6 months' duration, (3) acute lower limb musculoskeletal injury within the past 6 months, and (4) neurological or vestibular disorders.

### 2.2 Clinical Data

Before surgery, demographic and clinical information—including age, sex, height, weight, body mass index (BMI), and visual analog scale (VAS) scores—was collected. VAS was used to measure the severity of pain experienced by the participants. Ankle function was assessed using the American Orthopedic Foot and Ankle Society (AOFAS) Ankle‐Hindfoot Scale.

The anterior drawer test was conducted by an orthopedics (FC) with more than 10 years of experience in foot and ankle surgery. A positive result was defined as a substantial anterior displacement of the talus in comparison to the contralateral healthy side during the examination.

Based on current evidence that syndesmotic injuries can increase ankle rotation and that calcaneofibular ligament (CFL) injuries may affect subtalar joint motion [24, 25, 26], the presence of these injuries was recorded during arthroscopy to account for their potential impact on rotational stability.

### 2.3 Imaging Measurements

**FIGURE 1:**
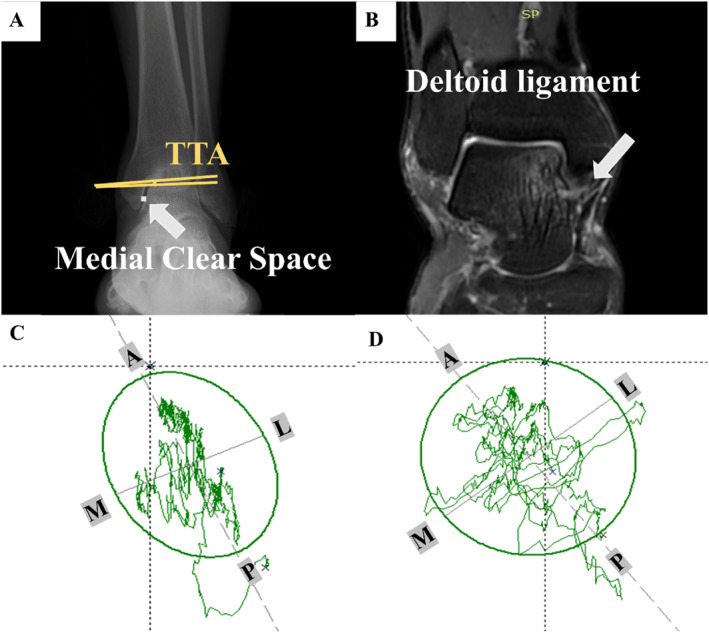
Diagnostic imaging and COP indicators. (A) Radiographic indicators: TTA and MCS; The TTA > 0 indicates varus and < 0 indicates valgus; (B) Magnetic resonance imaging scan: Grade 0 denotes an intact ligament. Grade I is defined by periligamentous edema; grade II by partial tearing with laxity, irregularity, or discontinuity and hyperintensity; and grade III by a complete ligament rupture [27]; (C and D) Illustration of the center of pressure indicator. A, anterior; L, lateral; M, medial; P, posterior.

All radiographic imaging was performed by a radiologist to reduce variability. Weight‐bearing anteroposterior X‐rays (Siemens Healthcare, Milan, Italy) were used to measure the talar tilt angle (TTA) and medial clear space (MCS) (Figure 1A) [11, 28, 29, 30]. MRI (Philips GE750 3.0 T, Amsterdam, Netherlands) was independently evaluated by two foot and ankle surgeons, each with over 8 years of experience, to assess the DL (Figure 1B) [2, 27, 31]. DL injury on MRI was graded as follows:

- Grade 0: Intact ligament
- Grade I: Periligamentous edema
- Grade II: Partial tearing with laxity, irregularity, or discontinuity and hyperintensity
- Grade III: Complete ligament rupture

### 2.4 Experimental Equipment

Two primary systems were used for the postural control assessments:

1. Sensory Organization Test (SOT)
  Conducted using the NeuroCom Balance Assessment System (Natus Medical, Seattle, USA), considered the gold standard for postural control [32]. The test is designed to evaluate a participant's ability to maintain balance under various controlled sensory conditions (Table 1).
2. Center of Pressure (COP) Analysis
  Performed using the FreeMED plantar pressure system (Sensor Medica, Rome, Italy) to measure COP sway during single‐leg stance.

**TABLE 1:** Sensory organization testing six environment configurations [19].

Condition	Eyes	Surface	Surround	Interference	Anticipated response
1	Open	Fixed	Fixed	Vision	Somatosensory
2	Closed	Fixed	Fixed	Vision	Somatosensory
3	Open	Fixed	Sway referenced	Vision	Somatosensory
4	Open	Sway referenced	Fixed	Somatosensory	Vision, vestibular
5	Closed	Sway referenced	Fixed	Somatosensory, vision	Vestibular
6	Open	Sway referenced	Sway referenced	Somatosensory, vision	Vestibular

### 2.5 Experimental Procedure and Testing Parameters

All participants underwent standardized postural control testing while wearing a safety harness to prevent falls. During SOT, the main outcome measures were somatosensation (SOM), vision (VIS), and vestibular (VEST) scores, with higher values indicating better postural control.

Single‐leg stance COP tests were performed under eyes‐open (EO) and eyes‐closed (EC) conditions, each lasting 10 s. During EO trials, participants focused on a red dot at eye level, 65 cm away, to reduce visual distractions. COP data were filtered using a low‐pass filter with a cutoff frequency of 5 Hz, minimizing noise while preserving critical sway information [33]. The measured COP parameters included:

- Sway length
- Sway area
- Anterior–posterior (AP) sway distance
- Medial–lateral (ML) sway distance
- Sway velocity
- Root mean square (RMS) of AP and ML sway distances

Lower COP sway values reflect better postural control [34]. Ratios, such as **Sway‐ML/AP**, **Sway‐ML/Area**, **Sway‐AP/Area**, and **RMS‐ML/AP,** were also calculated to characterize the swing pattern.

### 2.6 Study Procedure

All patients with CAI underwent preoperative postural control testing. During surgery, arthroscopy was used to evaluate the ATFL, CFL, and deep DL. Based on arthroscopic findings, patients were categorized into either the CAI group (no DL injury) or the RAI group (presence of DL injury).

### 2.7 Arthroscopic Examination

**FIGURE 2:**
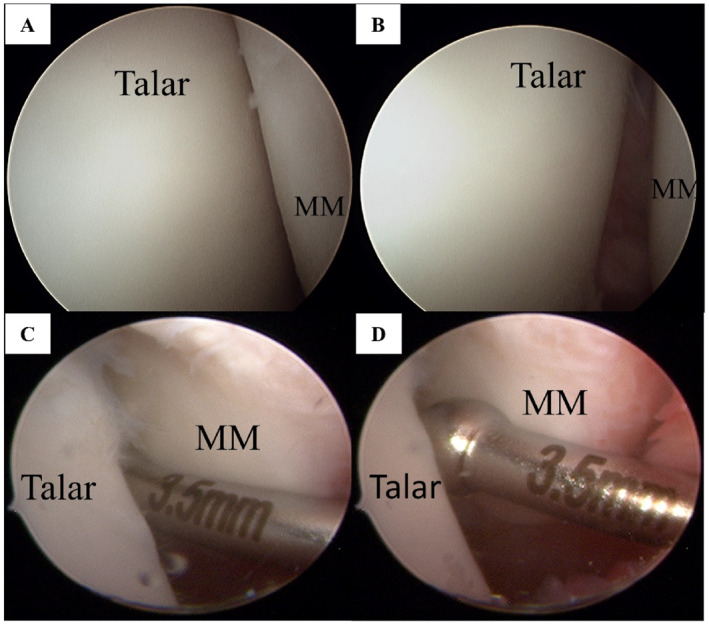
Arthroscopic view of the medial gutter (A–D). (A) “Open‐book” appearance with gapping in the medial gutter between the talar dome and the medial malleolus; (B) Another view confirming the “open‐book” finding; (C) Introduction of a 3.5‐mm probe into the medial mortise without traction, indicating chronic medial (deltoid) ligament abnormality; (D) Close‐up showing the 3.5‐mm probe passing into the medial gutter/mortise. MM: malleolus medialis.

Arthroscopy, considered the current standard for diagnosing DL injuries, was used to confirm “open book” (Figure 2A,B) [1, 8, 10, 31]. Examination of the DL by arthroscopy using current published techniques [1, 2]. In the present study, chronic medial ligament abnormality was defined as enough to introduce a 3.5 mm probe into medial mortise without using a traction device, based on Hintermann's classification (Figure 2C,D) [5, 6, 35].

### 2.8 Blinding

The study employed a blinded methodology with three independent orthopedists conducting the tests. Group assignment was concealed until the completion of data collection and the start of statistical analysis.

### 2.9 Statistical Analysis

Sample size was calculated using G*Power (HHU Düsseldorf, Germany) [36]. Based on preliminary data from six subjects in each group, with *α* = 0.05, power (1 − *β*) = 0.8, and an effect size of 1.36, a total of 20 participants and 10 in each group was deemed sufficient.

Data were analyzed using SPSS 26.0 (IBM, USA). Normality was assessed by the Shapiro–Wilk test. Normally distributed data are presented as mean ± standard deviation (Mean ± SD) and were compared using independent‐sample *t*‐tests. Non‐normally distributed variables are presented as median [Q1 and Q3] and were compared using the Mann–Whitney *U* test. Categorical data are reported as frequencies (percentages) and were evaluated via *χ* ^2^ tests. Statistical significance was set at *p* < 0.05.

The inter‐rater reliability for the MRI grading was assessed by computing a weighted Kappa statistic using Python (version 3.9.7) with the scikit‐learn library [37]. Linear weighting was applied to measure agreement between the two observers.

Spearman correlation analyses were performed to evaluate relationships between key variables and RAI status. Receiver operating characteristic (ROC) curves were plotted to determine the area under the curve (AUC), optimal cutoff values, and associated sensitivity and specificity. AUC interpretation was as follows [16]:

- AUC ≤ 0.5, no diagnostic value
- 0.5 < AUC ≤ 0.7, low diagnostic value
- 0.7 < AUC ≤ 0.9, moderate diagnostic value
- AUC > 0.9, high diagnostic value

## 3 Results

### 3.1 Demographic and Imaging Characteristics

**FIGURE 3:**
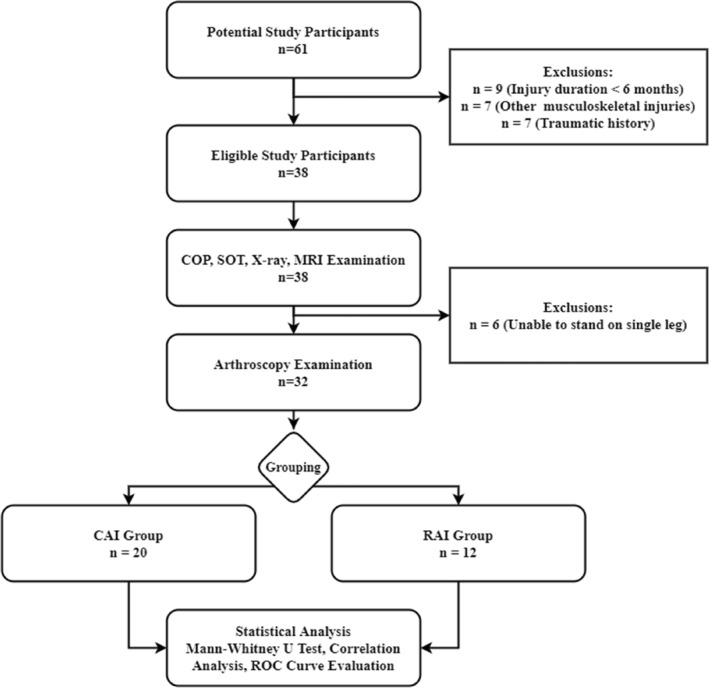
Flowchart of the study.

A total of 20 patient with CAIs and 12 patient with RAI were enrolled as illustrated in Figure 3. Baseline characteristics (age, sex, BMI, VAS, and AOFAS) and complications (osteochondral lesions, syndesmotic injuries, and calcaneofibular injuries) showed no significant differences between the two groups (Table 2). In the RAI group, arthroscopy confirmed a DL “open book” sign.

**TABLE 2:** Basic and radiographic information of two groups.

Index	CAI	RAI	*p* value
	*n* = 20	*n* = 12	
Basic information			
Age, *y*	41.65 ± 12.31	36.75 ± 11.25	0.270
Male/female, *n*	10/10	5/7	0.647
BMI (kg/m^2^)	24.52 ± 3.27	25.07 ± 2.64	0.625
VAS (scores)	3.35 ± 1.09	3.83 ± 1.12	0.238
AOFAS (scores)	67.1 ± 7.66	68.83 ± 8.17	0.550
OLT, *n*	13 (65%)	9 (75%)	0.555
Syndesmosis, *n*	8 (40%)	8 (66.7%)	0.144
CFL, *n*	2 (10%)	0 (0%)	0.258
Medial tenderness	5 (25%)	7 (58.33%)	0.059
X‐ray			
MCS (mm)	2.02 ± 0.67	2.49 ± 1.31	0.191
Talar tilt	−0.3 (−0.88, 1.25)	−0.65 (−1.38, 1.1)	0.533
MRI			
Deltoid ligament	1 (0–1)	2 (2–3)	0.001**

*Note:* Baseline data were compared between the CAI and RAI groups. Normal distribution data were mean (±) SD; non‐normal distribution *M* (Q1, Q3).

Abbreviations: CFL, calcaneofibular ligament injuries; OLT, osteochondral lesions of the talus; syndesmosis, syndesmotic injuries.

** *p* < 0.01.

X‐ray measurements (MCS and TTA) did not differ significantly between the groups, whereas MRI classification was significantly higher in the RAI group (*p* < 0.001). Inter‐rater reliability for MRI grading was substantial (Table 3; weighted *κ* = 0.612).

**TABLE 3:** Assessment of inter‐rater reliability for deltoid ligament grading between two evaluators (weighted kappa).

	Grade 0	Grade 1	Grade 2	Grade 3	Total	Percent (%)
Grade 0	5	1	0	0	6	18.75
Grade 1	2	2	7	0	11	34.38
Grade 2	0	1	4	2	7	21.88
Grade 3	0	0	2	6	8	25
Total	7	4	13	8	32	100
Percent (%)	21.88	12.5	40.63	25	100	—

*Note:* Rows correspond to grades assigned by Evaluator 1; columns correspond to grades assigned by Evaluator 2. Grey‐shaded cells indicate exact agreement (main diagonal); grey‐shaded “Total” cells are marginal totals.

### 3.2 Postural Control Parameters

**FIGURE 4:**
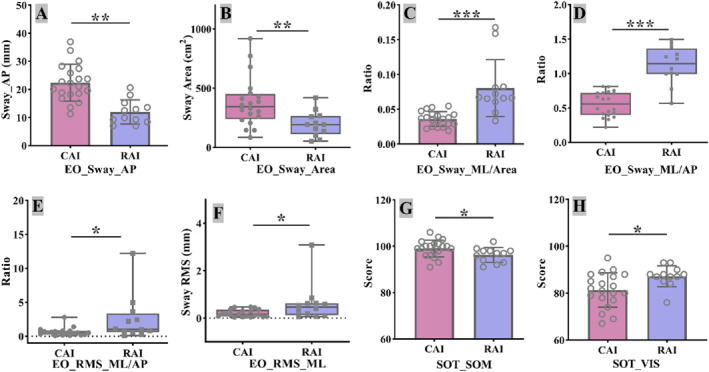
Comparison between groups of RAI and CAI in postural control index. (A) EO_Sway_AP (AP sway distance, eyes open); (B) EO_Sway_Area (total sway area, eyes open); (C) EO_Sway_ML/Area (ratio of ML sway to sway area); (D) EO_Sway_ML/AP (ratio of ML to AP sway distance); (E) EO_RMS_ML/AP (ratio of ML to AP COP RMS); (F) EO_RMS_ML (ML COP RMS, eyes open); (G) SOT_SOM (somatosensory score); (H) SOT_VIS (visual score). AP, anterior–posterior sway distance; CAI, chronic ankle instability; COP, center of pressure test; EC, eyes closed; EO, eyes open; ML, medial‐lateral sway distance; RAI, rotational ankle instability; RMS, root mean square; SOM, somatosensory; SOT, sensory organization test; VIS, visual. *p < 0.05; **p < 0.01; ***p < 0.001.

In the EO condition, the RAI group exhibited significantly lower AP sway distance and total sway area (both *p* < 0.01 and Figure 4A,B) but showed greater coronal‐plane instability. Specifically, coronal sway energy and coronal sway ratios (Sway‐ML/AP and RMS‐ML/AP) were significantly higher in the RAI group (all *p* < 0.05 and Figure 4C–F).

For the Sensory Organization Test (SOT), the RAI group had more pronounced proprioceptive deficits (*p* < 0.05 and Figure 4G) but higher visual scores (*p* < 0.05 and Figure 4H), suggesting greater reliance on visual input for balance.

### 3.3 Correlation Analysis

**FIGURE 5:**
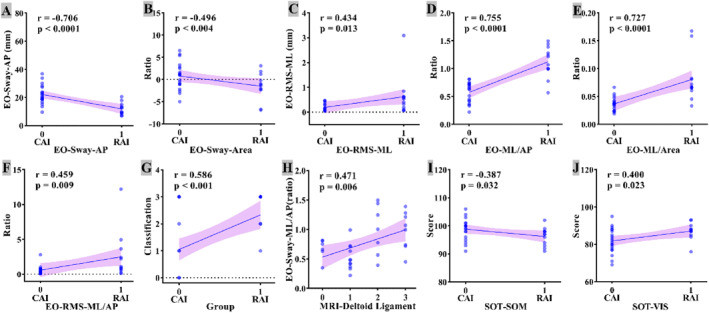
Correlation analysis between RAI and significant parameters (A–J). (A) EO_Sway_AP (AP COP sway distance, eyes open) versus group (0 = CAI, 1 = RAI); (B) EO_Sway_Area (total COP sway area, eyes open) versus group; (C) EO_RMS_ML (ML COP RMS, eyes open) versus group; (D) EO_Sway_ML/AP (ratio of ML to AP sway distance) versus group; (E) EO_Sway_ML/Area (ratio of ML sway to sway area) versus group; (F) EO_RMS_ML/AP (ratio of ML to AP COP RMS) versus group; (G) Classification score (probability of RAI) versus group; (H) EO_Sway_ML/AP versus MRI‐assessed deltoid‐ligament grade; (I) SOT_SOM score versus group; (J) SOT_VIS score versus group. AP, anterior–posterior sway distance; CAI, chronic ankle instability; COP, center of pressure; EO, eyes open; ML, medial‐lateral sway distance; MRI, magnetic resonance imaging; RAI, rotational ankle instability; RMS, root mean square; SOM, somatosensory; SOT, sensory organization test; VIS, visual.

Among the raw COP parameters, only AP sway (COP‐AP, *r* = −0.706, and *p* < 0.0001), total sway area (COP‐Area, *r* = −0.496, and *p* = 0.004), and RMS‐ML (*r* = 0.434 and *p* = 0.013) correlated significantly with RAI (Figure 5A–C). ***Coronal ratios*** (ML/AP, ML/Area, and RMS‐ML/AP) showed stronger positive correlations with RAI (up to *r* = 0.755, *p* < 0.0001, and Figure 5D–F) and were also associated with higher MRI grades of DL injury (Figure 5G,H). SOT proprioception scores were negatively correlated with RAI (*r* = −0.387 and *p* = 0.032), whereas visual scores correlated positively (*r* = 0.400, *p* = 0.023, and Figure 5I,J).

### 3.4 ROC Analysis for RAI Diagnosis Based on Significant Indicators

**FIGURE 6:**
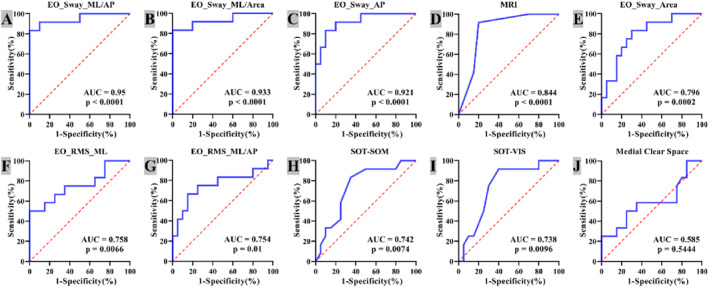
Receiver operating characteristic (ROC) curve analysis for distinguishing RAI using postural control, X‐ray, and MRI parameters (A‐J). (A) ROC of EO_Sway_ML/AP (ratio of mediolateral to anteroposterior sway distance); (B) ROC of EO_Sway_ML/Area (ratio of mediolateral sway to sway area); (C) ROC of EO_Sway_AP (AP sway distance); (D) ROC of MRI—deltoid‐ligament grade; (E) ROC of EO_Sway_Area (total sway area); (F) ROC of EO_RMS_ML (ML COP RMS); (G) ROC of EO_RMS_ML/AP (ratio of ML to AP COP RMS); (H) ROC of SOT_SOM (somatosensory score); (I) ROC of SOT_VIS (visual score); (J) ROC of medial clear space (radiograph). AP, anterior–posterior sway distance; AUC, area under the curve; EO, eyes open; ML, medial‐lateral sway distance; RMS, root mean square; SOM, somatosensory; SOT, sensory organization test; VIS, visual.

ROC curves for the most significant indicators revealed that ***COP‐ML/AP*** demonstrated the highest diagnostic accuracy (AUC = 0.95 and *p* < 0.0001), with an optimal cutoff of 0.813 yielding 83.33% sensitivity and 100% specificity (Figure 6A). COP‐ML/Area and COP‐Sway‐AP also showed high diagnostic value (AUC = 0.933 and 0.921; Figure 6B,C), whereas MRI, COP‐Area, RMS‐ML/AP, SOM, and VIS exhibited moderate accuracy (AUC 0.7–0.9; Figure 6D–I; *p* < 0.05). In contrast, MCS provided poor diagnostic discrimination (AUC < 0.70 and *p* > 0.05).

### 3.5 Diagnostic Accuracy of Coronal Instability in COP

Designating an EO‐COP‐ML/AP ratio > 0.813 as a positive indicator of coronal instability yielded a positive predictive value of 100% and a negative predictive value of 90.9%. The likelihood ratio for a positive test was 0% and 17% for a negative test (95% CI: 4.7%–59%). These results underscore the potential of coronal‐plane instability metrics to serve as a robust noninvasive diagnostic tool for identifying RAI in CAI populations (Tables 4 and 5).

**TABLE 4:** Diagnostic results of the coronal instability compared to arthroscopy.

		Arthroscopy		Totals
		Positive	Negative	
Coronal instability	Positive	10	1	11
	Negative	2	19	21
Totals		12	20	32

**TABLE 5:** Diagnostic value of each index for rotational ankle injury.

Index			AUC (95% Cl)	Cut‐off	Sensitivity	Specificity	PLR	NLR	PPV	NPV	*p* value
COP	Length (mm)	EO	0.617 (0.429–0.782)	≤ 80.37	50.0	90.00	5.00	0.56	75.0	75.0	0.3226
		EC	0.550 (0.365–0.750)	> 196.45	41.67	80.00	2.08	0.73	55.6	69.6	0.6428
	Area (cm^2^)	EO	0.794 (0.617–0.917)	≤ 267.1	83.33	70.00	2.78	0.24	62.5	87.5	0.0002***
		EC	0.583 (0.396–0.754)	> 327.17	91.67	35.00	1.41	0.24	45.8	87.5	0.1490
	Speed (mm/s)	EO	0.625 (0.437–0.789)	≤ 7.45	58.33	85.00	3.89	0.49	70.0	77.3	0.2917
		EC	0.579 (0.392–0.751)	> 18.08	33.33	90.00	3.33	0.74	66.7	69.2	0.4625
	M‐L (mm)	EO	0.508 (0.326–0.689)	> 13.98	16.67	55.00	0.37	1.52	18.2	52.4	0.9373
		EC	0.504 (0.323–0.685)	> 19.11	25.00	55.00	0.56	1.36	25.0	55.0	0.9679
	A‐P (mm)	EO	0.912 (0.769–0.986)	≤ 15.18	83.33	90.00	8.33	0.19	83.3	90.0	0.0001***
		EC	0.571 (0.385–0.744)	> 30.72	33.33	95.00	6.67	0.7	80.0	70.4	0.5294
	RMS‐ML (mm)	EO	0.758 (0.575–0.891)	> 0.471	50.0	100.0	NA	0.50	100.0	74.1	0.0066**
		EC	0.529 (0.346–0.707)	≤ 0.11	16.67	100	NA	0.83	100	66.7	0.7929
	RMS‐AP (mm)	EO	0.546 (0.361–0.722)	≤ 0.28	66.67	60.00	1.67	0.56	50.0	75.0	0.6591
		EC	0.510 (0.328–0.712)	> 0.957	8.33	75.00	0.33	1.22	16.7	57.7	0.9201
COP (ratio)	ML/AP	EO	0.950 (0.810–0.996)	> 0.813	83.33	100	NA	0.17	100	90.9	0.0001***
		EC	0.517 (0.334–0.696)	≤ 0.797	41.67	35.00	0.64	1.67	27.8	50.0	0.8826
	ML/area	EO	0.933 (0.786–0.991)	> 0.055	83.33	100	0	0.17	100	90.9	0.0001***
		EC	0.600 (0.412–0.768)	≤ 0.023	25.00	95.00	5.00	0.79	75.0	67.9	0.3392
	AP/area	EO	0.525 (0.342–0.704)	≤ 0.068	66.67	55.00	1.48	0.61	47.1	73.3	0.8130
		EC	0.500 (0.319–0.681)	> 0.065	8.33	65.00	0.24	1.41	12.5	54.2	1
	RMS‐ML/AP	EO	0.754 (0.570–0.888)	> 0.791	66.67	85.00	4.44	0.39	72.7	81.0	0.0101*
		EC	0.542 (0.357–0.718)	≤ 1.118	83.33	40.00	1.39	0.42	45.5	80.0	0.7028
SOT	SOM		0.742 (0.557–0.879)	≤ 98	83.33	65.00	2.38	0.26	58.8	86.7	0.0074**
	VIS		0.738 (0.552–0.885)	> 83	91.67	60.00	2.29	0.14	57.9	92.3	0.0096**
	VEST		0.679 (0.491–0.832)	> 72	75.0	65.00	2.14	0.38	56.2	81.2	0.0723
X‐ray	MCS		0.585 (0.398–0.756)	> 2.29	50.00	75.00	2.0	0.67	54.5	71.4	0.4471
	Talar tilt		0.567 (0.381–0.740)	≤ −1.1	50.00	80.00	2.5	0.63	60.0	72.7	0.5444
MRI	Deltoid		0.844 (0.672–0.947)	> 1	91.67	80.00	4.58	0.10	73.3	94.1	0.0001***

Abbreviations: COP, center of pressure; EC, eyes close; EO, eyes open; NA, missing value; NLR, negative likelihood ratios; NPV, negative predictive value; PLR, positive likelihood ratios; PPV, positive predictive value; ratio, after‐test/before‐test.

* *p* < 0.05.

** *p* < 0.01.

*** *p* < 0.001.

## 4 Discussion

This study highlights the diagnostic potential of postural control measures, especially the COP‐ML/AP ratio, for identifying RAI. Our results demonstrate that patients with RAI exhibit marked coronal‐plane instability, with a ratio exceeding 0.81 under eyes‐open conditions emerging as a clear quantifiable threshold.

### 4.1 Traditional Imaging

Traditional imaging modalities, such as X‐rays and MRI, have inherent limitations in predicting functional and dynamic instabilities, largely due to their static nature. X‐rays are frequently used to assess the MCS for signs of DL injury [10]. Leith suggested that manual eversion and weight‐bearing during X‐rays could improve sensitivity and specificity for DL injuries [28, 30]. However, our findings did not support the use of MCS and TAA in distinguishing between RAI and CAI. Due to positional and rotational inconsistencies that affect X‐ray imaging accuracy [38]. Therefore, although X‐rays may play a role in early screening, they are insufficient for reliably diagnosing RAI.

MRI is a well‐established tool for soft tissue assessment, particularly for identifying ligament injuries. Chun reported high concordance between MRI and arthroscopic findings in diagnosing DL injuries [5]. Our study supports these findings confirming the high sensitivity of MRI in detecting DL damage. However, lower specificity of MRI suggests its role may be more suited for initial screening rather than for precise grading of ligament injuries and diagnose it [4, 6, 39]. Additionally, Kumar noted that in cases of CAI, MRI alone often struggles to accurately assess CFL damage, especially when relying on signal intensity, making weight‐bearing radiographs a more reliable option in anesthetized cases [12]. Hence, in chronic cases or partial tears, MRI findings may not align completely with arthroscopic evaluations, which limits diagnostic reliability [40].

### 4.2 Postural Control and Coronal Instability

In contrast to static assessments, postural control testing directly measures COP sway, providing a dynamic perspective on ankle stability [16]. We observed that patients with RAI exhibit an atypical “circular” COP swing pattern, suggesting increased torsional stress within the ankle‐tibia complex [41]. This deviates from the more elliptical anterior–posterior–dominant sway commonly seen in healthy or purely lateral instability. According to current evidence, the balance strategy of the RAI may involve a complex pattern of lower limb torsion in the ankle and tibia [1, 4, 10].

Notably, patients with RAI demonstrated lower proprioceptive scores but higher visual scores in the SOT. The SOT measures balance maintenance while standing, incorporating multiple sensory disruptions. Higher scores indicate less COP sway. This suggests “less sway and better balance,” which aligns with their visual compensation strategy. Interestingly, patients with RAI showed small sway area under the EO condition instead of EC condition during single‐leg posture. At first glance, this may seem contradictory, but it likely reflects a distinct balance strategy.

However, patients with RAI exhibited a greater ratio of sway in the coronal plane. This increased ratio of ML may indicate compensatory torsional adjustments in the lower limb to maintain balance. These adjustments could involve the foot arch, talus, tibia, or even the entire lower limb, resulting in rotational movement of the ankle joint. This, in turn, triggers a chain reaction, increasing muscular activity to stabilize the body in a conservative manner [42, 43, 44].

Moreover, the DL play a critical role in ankle stability and function [10, 15]. RAI is associated with multiple‐ligament damage, which leads to structural instability and impaired proprioception [45]. The visual advantage observed under the EO condition may suggest that, despite visual input, multiple ligament injuries contribute to structural instability and proprioceptive receptor damage, resulting in a loss of proprioceptive feedback. Although visual input can partially compensate for proprioceptive loss to maintain balance, this compensatory ability is likely limited.

It is important to note that the SOT uses a force plate that cannot capture neural signals from the lower limb muscles or kinematic data above the plate [32, 46]. As such, further studies are needed to explore the underlying causes of these observed differences. Future research could incorporate eye trackers and brain function testing devices as recent studies have identified deficits in movement‐related brain regions in patients with CAI, potentially linked to more complex neuromuscular control mechanisms [47].

### 4.3 Clinical Implications

Our discovery of a COP‐ML/AP ratio > 0.81 in the EO condition as a strong indicator of RAI provides a highly sensitive and specific measure for clinical evaluation. By dividing the ML sway distance by the AP sway distance in the filtered COP data, this result can be obtained. Furthermore, after normalizing ML sway to AP sway, this metric partly accounts for individual anatomical and biomechanical variations. Although arthroscopy is considered the current standard for directly visualizing intra‐articular pathology and confirming RAI (“open book” sign), its invasive nature limits widespread routine use. Consequently, postural control tests may function as an adjunct to imaging for detecting RAI.

### 4.4 Limitations

Several limitations must be noted. First, our sample size was relatively small, though statistical power exceeded 0.8. Second, we did not arthroscopically evaluate superficial DL injuries, focusing primarily on deep DL pathology associated with more pronounced rotational instability [1, 2, 15]. Third, although CT‐based HU measurements have been proposed to evaluate ligament degeneration, technical challenges in delineating standard HU value prevented their inclusion here [7]. Nonetheless, although these functional assessments provide valuable insights into ligament stability, they cannot fully replace structural imaging when evaluating multiple or complex ligament injuries [1, 4, 48]. Integrating postural control data with MRI findings may offer a more comprehensive clinical perspective, ultimately enhancing diagnostic confidence and informing more targeted interventions. Moreover, although there was no statistical difference between the two groups in terms of syndesmotic injury, two‐thirds of the RAI group also had syndesmotic injury. Future studies should further explore the interaction between the two and its impact on treatment strategies. Finally, our cross‐sectional design precludes an assessment of long‐term outcomes, and future longitudinal studies are necessary to determine whether coronal instability on postural testing correlates with clinical progression or surgical outcomes over time.

## 5 Conclusion

Our findings suggest that postural control testing—particularly the COP‐ML/AP ratio exceeding 0.81 in the eyes‐open condition—could be a valuable noninvasive adjunct in evaluating rotational ankle instability. By capturing coronal‐plane sway deviations, this functional measure provides a novel perspective that complement traditional imaging‐based assessments of ankle stability, offering clinicians additional insights that are not readily available through conventional modalities.

## Author Contributions

**Nan Mei:** conceptualization, study design, data analysis, writing – original draft, writing – review and editing. **Zhende Jiang:** conceptualization, data collection, writing – review and editing. **Zhuan Zhong:** data collection, writing – review and editing. **Yaokuan Ruan:** data collection, writing – review and editing. **Hengyu Liu:** data collection, writing – review and editing. **Hiroaki Kurokawa:** data analysis, writing – review and editing. **Takuma Miyamoto:** data analysis, writing – review and editing. **Akira Taniguchi:** data analysis, writing – review and editing. **Yasuhito Tanaka:** data analysis, writing – review and editing. **Fei Chang:** funding acquisition, project administration, writing – review and editing.

## Ethics Statement

The study was approved by the Ethics Committee of the Second Hospital of Jilin University (No. 2021130).

## Consent

All participants gave their written informed consent to publish the obtained data of the current study. Informed consent was obtained from all individual participants included in the study.

## Conflicts of Interest

The authors declare no conflicts of interest.

## Data Availability

Data are available from the corresponding author under reasonable request.
